# Clinical and Mutational Analysis of the* GCDH* Gene in Malaysian Patients with Glutaric Aciduria Type 1

**DOI:** 10.1155/2016/4074365

**Published:** 2016-09-08

**Authors:** Siti Aishah Abdul Wahab, Yusnita Yakob, Nor Azimah Abdul Azize, Zabedah Md Yunus, Leong Huey Yin, Mohd Khairul Nizam Mohd Khalid, Ngu Lock Hock

**Affiliations:** ^1^Molecular Diagnostics and Protein Unit, Institute for Medical Research, Kuala Lumpur, Malaysia; ^2^Biochemistry Unit, Institute for Medical Research, Kuala Lumpur, Malaysia; ^3^Department of Genetics, Hospital Kuala Lumpur, Kuala Lumpur, Malaysia

## Abstract

Glutaric aciduria type 1 (GA1) is an autosomal recessive metabolic disorder caused by deficiency of glutaryl-CoA dehydrogenase enzyme encoded by the* GCDH* gene. In this study, we presented the clinical and molecular findings of seven GA1 patients in Malaysia. All the patients were symptomatic from infancy and diagnosed clinically from large excretion of glutaric and 3-hydroxyglutaric acids. Bidirectional sequencing of the* GCDH* gene revealed ten mutations, three of which were novel (Gln76Pro, Glu131Val, and Gly390Trp). The spectrum of mutations included eight missense mutations, a nonsense mutation, and a splice site mutation. Two mutations (Gln76Pro and Arg386Gln) were homozygous in two patients with parental consanguinity. All mutations were predicted to be disease causing by MutationTaster2. In conclusion, this is the first report of both clinical and molecular aspects of GA1 in Malaysian patients. Despite the lack of genotype and phenotype correlation, early diagnosis and timely treatment remained the most important determinant of patient outcome.

## 1. Introduction

Glutaric aciduria type 1 (GA1, OMIM #231670) is an autosomal recessive disorder of lysine, hydroxylysine, and tryptophan caused by deficiency of glutaryl-CoA dehydrogenase (GCDH, EC 1.3.8.6) enzyme. The disease was first described in 1975 [1] and has an estimated prevalence of 1 in 100,000 newborns [2]. Defective GCDH enzyme results in accumulation of the glutaric acid (GA), 3-hydroxyglutaric acid (3-OH-GA), glutaconic acid, and glutarylcarnitine (C5DC). These can be detected in body fluids (urine, plasma, and CSF) and tissues by gas chromatography/mass spectrometry (GC/MS) or electrospray-ionization tandem mass spectrometry (MS/MS) [3, 4].

Patients often present macrocephaly at birth, accompanied by “soft” neurological symptoms of hypotonia, irritability, and jitteriness [5]. About 75% of the patients then suffer an acute encephalopathic crisis, usually associated with an upper respiratory and/or gastrointestinal infection, immunization, or surgical intervention between the ages of 2 and 37 months [5, 6]. After recovery, the patients lost motor skills and function and a severe dystonic-dyskinetic syndrome in children with relatively well-preserved intellectual functions and a prominent forehead may be recognized [5]. If by this stage the underlying metabolic disorder remains undiagnosed, cerebral atrophy develops with clinical findings of pyramidal tract signs and mental retardation [5]. Treatment consisting of lysine-restricted diet using natural protein with a low lysine content and appropriate amino acid supplements (lysine-free, tryptophan-reduced) was effective especially if administered in presymptomatically diagnosed patients [6].

GCDH enzyme is encoded by* GCDH* gene which is located on chromosome 19p13.2 spanning about ~7 kb region and contains 12 exons (transcript ID ENST00000222214) [7, 8]. To date, close to 200 pathogenic mutations have been reported in the Human Gene Mutation Database (HGMD) with missense mutation being the most common type (http://www.hgmd.cf.ac.uk/ac/index.php). In specific ethnic groups, a few common mutations were prevalent such as Ala421Val in the Amish [8], Arg402Trp in the Europeans [9], and IVS10-2A>C in the Chinese [10]. In Malaysia, GA1 is the second most common organic acidurias, constituting about 7.2% of inborn errors of metabolism (IEM) diagnosed at the Institute for Medical Research (IMR) from year 1999 to year 2005 [11]. However, mutational analysis of GA1 patients from Malaysia has never been reported. Therefore, in this study we investigated the clinical and molecular aspects of seven Malaysian patients with GA1.

## 2. Materials and Methods

### 2.1. Patients Enrolment

Seven patients with GA1 were recruited at the Metabolic Clinic of Hospital Kuala Lumpur (HKL). Diagnosis was established based upon clinical presentations, increased excretion of GA and 3-OH-GA in urine organic acid analysis and tandem mass spectrometry analysis of C5DC in dried blood spots (DBS). Parents of the affected child were guided to sign a consent form for genetic testing. Parental consanguinity was observed in patients 5 and 7 (Table 1). Sample from patient 3 was taken when the patient was 8 years old upon visit to the Metabolic Clinic; however, the patient has passed away of pneumonia at 11 years of age.

Clinical outcomes of patients with GA1 were evaluated based on the motor and speech disability assessment developed by Kyllerman et al. [15]. Briefly, motor disability was scored as 1, 2, or 3 for mild, moderate, or severe motor dysfunction, respectively. Patients with severe motor dysfunction were wheelchair dependent and showed severe disability in everyday life [15] whereas mild motor disability is mild motor dysfunction and no disability in daily life. Speech was scored as 1, 2, or 3 depending on whether the patient is fluent, could express only single words, or has no speech at all, respectively [15].

### 2.2. Mutation Analysis

Approximately 5 to 10 mL of peripheral blood was collected from all seven patients in EDTA tubes and genomic DNA was isolated using the QIAcube (Qiagen, Hilden, Germany). Both the quantity and quality of extracted DNAs were measured using NanoDrop ND-1000 Spectrophotometer (NanoDrop, Wilmington, DE, USA). Primers were designed in-house to amplify all coding exons and flanking intronic sequences of* GCDH* gene. PCR was performed in a volume of 50 *μ*L containing 100 ng genomic DNA, 0.1 U* Taq* DNA polymerase (Fermentas), 1x of* Pfu* PCR buffer with MgSO_4_, 1 *μ*mol of each of the primers, and 0.2 mM of dNTP mix. Amplification was performed using touchdown PCR protocol as described previously [16].

The PCR products were purified using the QIAquick PCR Purification kit (Qiagen, Hilden, Germany) according to manufacturer's instructions. Cycle sequencing was performed using the BigDye® Terminator cycle sequencing v3.1 chemistry (Applied Biosystems, Foster City, CA, USA) followed by purification using DyeEx 2.0 Spin Kit (Qiagen). Purified cycle sequencing products were dried on vacuum concentrator, resuspended in 10 *μ*L of Hi-Di formamide, and analyzed on the Applied Biosystems 3500 Genetic Analyzer. Sequencing results were aligned to the reference sequence of the* GCDH* gene (GenBank NM_000159.3) using SeqScape Software v3.0 (Applied Biosystems) to identify any DNA variants. All variants identified were annotated against publicly available databases such as the HGMD and dbSNP (http://www.ncbi.nlm.nih.gov/SNP/). The pathogenicity of novel DNA variants was evaluated by using MutationTaster2 software (http://www.mutationtaster.org/). DNA samples from 50 healthy individuals were tested for the presence of novel DNA variants identified in our patients.

## 3. Results

### 3.1. Clinical Findings

The clinical features of seven Malaysian GA1 patients are summarized in Table 1. All patients were symptomatic with onset of symptoms from 3 to 11 months of age (Mean: 5 months, SD ±2.8 months). Acute gastroenteritis was documented in patients 3 and 7 preceding insidious onset of neurological regression. Patients 1, 2, and 6 presented with acute encephalopathy. Patients 4 and 5 were diagnosed with bilateral subdural haemorrhage and subdural effusion, respectively, when investigated for macrocephaly. Both (patients 4 and 5) developed generalized dystonia following intracranial surgery.

The age at diagnosis varied considerably from 6 months to 13 years of age (Mean: 4 years, SD ±4.6 years). All patients had increased excretion of GA and 3-OH-GA in urine organic acid analysis. Patients 2, 5, and 6 showed mildly increased C5DC in DBS while patient 7 showed marked elevation. CT or MRI of the brain showed the typical widening of bilateral Sylvian fissures with frontotemporal atrophy in all patients except for patient 7 who has generalized cerebral atrophy. Upon diagnosis, all patients were treated on low protein diet supplemented with lysine/tryptophan-free synthetic formula and L-carnitine.

All patients except for patients 5 and 7 had severe motor disability and absence of speech. Patient 5 with moderate motor disability was able to walk with assistive device and attended normal school with educational support. Patient 7 has only mild motor impairment when last assessed at age 2.

### 3.2. Mutations in GCDH Gene

Ten mutations were identified in the seven patients, and three of them were novel (Table 1). These included eight missense mutations (Gln76Pro, Glu131Val, Ala298Thr, Gly354Ser, Arg355Cys, Arg386Gln, Gly390Trp, and Ala421Thr), a nonsense mutation (Arg128^*∗*^), and a splice site mutation (c.1244-2A>C). Three novel mutations (Gln76Pro, Glu131Val, and Gly390Trp) were not detected in our 50 healthy individuals tested excluding the probability of polymorphism. MutationTaster2 predicted all novel mutations as disease causing. The two patients with homozygous mutations (patients 5 and 7) were due to parental consanguinity. Two recurrent mutations (c.1244-2A>C and Arg355Cys) were identified in two unrelated patients.

## 4. Discussion

In this paper, we presented our findings on the clinical and molecular aspects of GA1 in seven patients from Malaysia. Clinically, our patients showed typical features of GA1 such as macrocephaly that was followed by acute encephalopathic crisis, resulting in neuroregression. Consequently, majority of our patients (5/7, 71.4%) suffered severe motor disability and absence of speech. Late diagnosis was a major issue contributing to the severe outcome observed in our patients with 57.1% (4/7) diagnosed after 1 year of onset of symptoms. If started before encephalopathic crisis, dietary treatment and L-carnitine supplementation can prevent such complications [6]. However, since clinical presentation was not specific before onset of encephalopathic crisis, MS/MS-based neonatal screening or DNA-based screening should be used to detect presymptomatic patients [6].

The spectrum of mutations identified in our patients was similar to previous report [17]. Two mutations, Arg128^*∗*^ and c.1244-2A>C, were predicted to abolish GCDH enzyme activity by producing a truncated protein. The Gly354Ser mutation was shown to abolish GCDH enzyme activity in cultured fibroblasts [13] while Ala298Thr mutation significantly reduced GCDH enzyme activity to 5–10% [18]. The Arg355Cys, Ala421Thr, and Gly390Trp mutations affected the same positions as Arg355His, Ala421Val, and Gly390Ala, respectively, all of which were reported to have no enzymatic activity by cultured fibroblasts or expression in* E. coli* [12, 13, 18].

Two recurrent mutations have been detected: Arg355Cys in patients 3 and 4 and c.1244-2A>C in patients 1 and 6. Both mutations were previously reported to be disease causing mutations as they abolished enzyme activity in* E. coli* and cultured fibroblast, respectively. Despite the absence of functional studies for novel mutations, Gln76Pro, Glu131Val, and Gly390Trp, they were predicted to be pathogenic based on MutationTaster2 and not present in our 100 normal alleles. Besides that, the amino acids of novel mutations are highly conserved among different species suggesting that the region plays an important role in GCDH activity.

Of the seven patients, two patients from consanguineous marriage (patients 5 and 7) were mildly affected with generally good prognosis. Patient 7 harboured changes from positively charged arginine to polar Glutamine (Arg386Gln) whereas patient 5 exhibited changes from polar hydrophilic Glutamine to nonpolar hydrophobic proline (Gln76Pro). Although MutationTaster2 predicted that both variants to be disease causing but it could not explain the mild phenotype in patients since very little correlation between genotype and phenotype was available in GA1 patients [13]. Even in siblings with the same mutations, there was a notable difference in the phenotypes, further indicating that genotype does not predict clinical outcome [17]. Therefore, better patient outcomes are not associated with genotypes but rather with early diagnosis and timely treatment [17].

In conclusion, we have characterized both the clinical and molecular aspects of GA1 in Malaysian patients. The setting up of biochemical and molecular testing for GA1 will hopefully allow for detection of presymptomatic patients that can be treated early. Functional studies especially for novel mutations should be considered to confirm the pathogenicity.

## Figures and Tables

**Table 1:** Summary of clinical and molecular characteristics of seven Malaysian patients with GA1.

Patient	1	2	3	4	5	6	7

Gender	Male	Male	Male	Male	Female	Female	Female

Race	Chinese	Chinese	Chinese	Chinese	Malay	Malay	Malay

Parental consanguinity	−	−	−	−	+	−	+

Age at onset	5 m	3 m	5 m	3 m	3 m	5 m	11 m

Age at diagnosis	6 y	13 y	8 m	7 m	5 y	6 m	2 y

Actual age	20 y	23 y	Died at 11 y	4 y	14 y	20 m	2 y

Findings at onset	Macrocephaly, seizures, irritable, dystonia, SDH	Macrocephaly, seizure, irritable, dystonia, SDE, hydrocephalus	Macrocephaly, neuroregression, SDH	Macrocephaly, SDH	Macrocephaly, SDH	Macrocephaly, seizure, irritable, dystonia, SDH	Macrocephaly, neuroregression, SDH

Precipitating illness	−	−	+	−	−	−	+

Dystonia	+	+	+	+	+	+	−

Motor disability	Severe	Severe	Severe	Severe	Moderate	Severe	Mild

Speech	Absent	Absent	Absent	Absent	Nearly fluent	Absent	Nearly fluent

CT/MRI brain findings	Typical^*∗*^	Typical^*∗*^, hydrocephalus	Typical^*∗*^	Typical^*∗*^, abnormal signal at putamen, cerebral atrophy	Typical^*∗*^, generalized cerebral atrophy	Typical^*∗*^	Generalized cerebral atrophy

Urine GA excretion	Increase	Increase	Increase	Increase	Increase	Increase	Increase

C5DC in DBS (ref. <0.22 *μ*mol/L)	ND	0.38	ND	ND	0.34	0.43	4.9

Nucleotide change	(1) c.892 G>A; (2) c.1244-2A>C	(1) c.1060G>A; (2) c.1168G>T	(1) c.1063 C>T; (2) c.1261 G>A	(1) c.382 C> T; (2) c.1063C>T	(1) c.227A>C (2) c.227A>C	(1) c.392A>T (2) c.1244-2A>C	(1) c.1157G>A (2) c.1157G>A

Protein change	p.Ala298Thr; Splice site	p.Gly354Ser; **p.Gly390Trp**	p.Arg355Cys; p.Ala421Thr	p.Arg128^*∗*^; p.Arg355Cys	**p.Gln76Pro** **p.Gln76Pro**	**p.Glu131Val** Splice site	p.Arg386Gln p.Arg386Gln

Reference	Goodman et al. (1998) [12] Tang et al. (2000) [10]	Schwartz et al. (1998) [13] This study	Goodman et al. (1998) [12] Goodman et al. (1998) [12]	Busquets et al. [14] Goodman et al. (1998) [12]	This study	This study Tang et al. (2000) [10]	Goodman et al. (1998) [12]

Pathogenicity prediction (MutationTaster2)	Disease causing (both)	Disease causing (both)	Disease causing (both)	Disease causing (both)	Disease causing	Disease causing (both)	Disease causing

“+” = present; “−” = absent; SDH = subdural haemorrhage; SDE = subdural effusion; GA = glutaric acids; ND = not determined; and Typical^*∗*^ = bilateral widening of the Sylvian fissures and atrophy over the frontotemporal regions.

Bold denotes novel mutation.

## Abbreviations

GA1: Glutaric aciduria type 1
GCDH: Glutaryl-CoA dehydrogenase
HGMD: Human Gene Mutation Database.
